# Fingolimod promotes peripheral nerve regeneration via modulation of lysophospholipid signaling

**DOI:** 10.1186/s12974-016-0612-9

**Published:** 2016-06-10

**Authors:** Fabian Szepanowski, Angelika Derksen, Irina Steiner, Gerd Meyer zu Hörste, Thomas Daldrup, Hans-Peter Hartung, Bernd C. Kieseier

**Affiliations:** Department of Neurology, Medical Faculty, Heinrich-Heine-University, Düsseldorf, Germany; Department of Forensic Toxicology, Institute of Legal Medicine, Medical Faculty, Heinrich-Heine-University, Düsseldorf, Germany; Evergrande Center for Immunologic Diseases, Ann Romney Center for Neurologic Diseases, Brigham and Women’s Hospital, Harvard Medical School, Boston, MA USA; Department of Neurology, University of Münster, Münster, Germany

**Keywords:** Fingolimod, Sphingosine-1-phosphate, Lysophosphatidic acid, PF-8380, Peripheral nerve regeneration

## Abstract

**Background:**

The lysophospholipids sphingosine-1-phosphate (S1P) and lysophosphatidic acid (LPA) are pleiotropic signaling molecules with a broad range of physiological functions. Targeting the S1P_1_ receptor on lymphocytes with the immunomodulatory drug fingolimod has proven effective in the treatment of multiple sclerosis. An emerging body of experimental evidence points to additional direct effects on cells of the central and peripheral nervous system. Furthermore, fingolimod has been reported to reduce LPA synthesis via inhibition of the lysophospholipase autotaxin. Here we investigated whether modulation of particular signaling aspects of S1P as well as LPA by fingolimod might propagate peripheral nerve regeneration in vivo and independent of its anti-inflammatory potency.

**Methods:**

Sciatic nerve crush was performed in wildtype C57BL/6, in immunodeficient *Rag1*^*−/−*^ and *Foxn1*^*−/−*^ mice. Analyses were based on walking track analysis and electrophysiology, histology, and cAMP formation. Quantification of different LPA species was performed by liquid chromatography coupled to tandem mass spectrometry. Furthermore, functional consequences of autotaxin inhibition by the specific inhibitor PF-8380 and the impact of fingolimod on early cytokine release in the injured sciatic nerve were investigated.

**Results:**

Clinical and electrophysiological measures indicated an improvement of nerve regeneration under fingolimod treatment that is partly independent of its anti-inflammatory properties. Fingolimod treatment correlated with a significant elevation of axonal cAMP, a crucial factor for axonal outgrowth. Additionally, fingolimod significantly reduced LPA levels in the injured nerve. PF-8380 treatment correlated with improved myelin thickness. Sciatic nerve cytokine levels were not found to be significantly altered by fingolimod treatment.

**Conclusions:**

Our findings provide in vivo evidence for direct effects of fingolimod on cells of the peripheral nervous system that may propagate nerve regeneration via a dual mode of action, differentially affecting axonal outgrowth and myelination by modulating relevant aspects of S1P and LPA signaling.

## Background

Lysophospholipids are metabolites of glycerophospholipids and sphingolipids that are commonly found as lipid constituents of cell membranes. Besides being a structural membrane component, certain members of the lysophospholipid family have considerable cell signaling properties [1]. Among these, sphingosine-1-phosphate (S1P) and lysophosphatidic acid (LPA) and their signaling pathways have been best characterized. Lysophospholipid signaling appears to have a large range of physiological and pathophysiological functions [2, 3] in the adult organism and also plays important roles during embryonic development, especially in the development of the nervous system and vascular development [4–6].

S1P acts as a ligand for five G-protein-coupled receptors [7–11], generally referred to as S1P_1–5_. Since S1P receptors couple to a variety of G-proteins, stimulation of S1P receptors can impact several signal transduction pathways and cellular processes. In contrast to S1P_2–5_, S1P_1_ is thought to exclusively couple with G_i/o_, affecting cyclic adenosine monophosphate (cAMP) levels and Ca^2+^ mobilization, among others [10, 12].

Fingolimod (also named FTY720) is a first-in-class S1P receptor agonist that has been approved for the treatment of remitting-relapsing multiple sclerosis due to its immunomodulatory properties [13]. Structurally, a sphingosine analogue, fingolimod becomes phosphorylated by sphingosine kinases [14] and binds to four of the five S1P receptors [15], except for S1P_2_. Its immunomodulatory effect is presumably based on its “functionally antagonistic” effect on the S1P_1_ receptor expressed on lymphocytes, causing S1P_1_ receptor internalization and subsequently abrogation of S1P_1_-mediated signaling. It thereby prevents lymphocyte egress [16, 17]. In addition, phosphorylated fingolimod (fingolimod-P) has been reported to act as an inhibitor of autotaxin, an ectonucleotide pyrophosphatase/phosphodiesterase with lysophospholipase D activity that generates LPA from more complex lysophospholipids [18]. LPA has been implicated in dorsal root demyelination after partial sciatic nerve injury, most likely by activation of LPA_1_ receptor signaling [19], and demyelination has been found to be significantly reduced in heterozygous autotaxin knockout mice [20]. Furthermore, traumatic brain injury has been reported to induce significant increases of various LPA species in cerebrospinal fluid already 3 hours after injury in mice, and administration of LPA-antibody improves brain tissue damage outcomes [21]. Additionally, recent publications point to a significant role of LPA and LPA_1_ receptor signaling in the pathophysiology of spinal cord injuries [22, 23].

As S1P receptors are known to be widely expressed in the nervous system [24], there is an emerging body of experimental evidence pointing to direct effects of fingolimod on cells of the central nervous system [25], including neuroprotection from excitotoxic death and the promotion of remyelination. Additionally, fingolimod has been reported to promote functional recovery in spinal cord injury models [26, 27]. However, the effects of fingolimod on cells of the peripheral nerve in vivo have not been investigated so far. To address the question whether fingolimod may promote peripheral axon regeneration or remyelination and to differentiate between potential direct PNS-specific and its established immunomodulatory effects, we performed sciatic nerve crush in wildtype C57BL/6 as well as in two immunodeficient mouse strains: *Rag1*^*−/−*^ mice lacking mature B- and T-lymphocytes and in athymic *Foxn1*^*−/−*^ mice devoid of T-lymphocytes. To further explore the role of LPA during sciatic nerve de- and regeneration as well as to distinguish the significance of S1P receptor modulation from LPA mediated effects, we treated animals with the autotaxin inhibitor PF-8380 [28] and assessed the effect of fingolimod on LPA formation after injury in sciatic nerve around the crush site.

## Methods

### Animals

*Rag1*^*−/−*^ mice (B6.129S7-*Rag1*^*tm1Mom*^/J) were obtained from Jackson Laboratories (Bar Harbor, ME, USA), *Foxn1*^*−/−*^ mice (B6.Cg/NTac-*Foxn1*^*nu*^) from Taconic (Hudson, NY, USA), and control C57BL/6 mice from Janvier Labs (Le Genest-Saint-Isle, France). Animal use and experiments were approved by local authorities (LANUV North Rhine-Westphalia, Germany).

### Sciatic nerve crush

Male, age-matched (3–4 months) wildtype C57BL/6, *Rag1*^*−/−*^, and *Foxn1*^*−/−*^ mice were anesthetized for surgery via intraperitoneal injection of a mixture of xylazine (Rompun; Bayer, Leverkusen, Germany) (10 mg/kg) and ketamine (Actavis, Munich, Germany) (100 mg/kg) and placed on a heating plate (37 °C) to maintain constant body temperature. The fur of the lower back was removed with an electric razor, and the skin was disinfected using 70 % ethanol. All instruments were sterilized. A small incision (1 cm) was made in the skin above the right hindlimb between the mm. gluteus maximus and biceps femoris. Opening the facial plane between both muscles revealed the sciatic nerve which was carefully lifted using bent forceps and crushed right before its distal branches using a non-serrated clamp at maximum intensity for 30 s. The nerve was replaced under the muscle, and the incision was closed using non-absorbable suture material. The contralateral nerve was left intact to serve as control.

### Administration of fingolimod and PF-8380

Mice received non-phosphorylated fingolimod (FTY720, Cayman Europe, Tallinn, Estonia) dissolved in a solution of 10 % DMSO in PBS (Sigma-Aldrich, Munich, Germany) via intraperitoneal injection at a concentration of 1 mg/kg once daily over the course of 16 days, starting 2 days before crush until 14 days post-crush. PF-8380 (Sigma-Aldrich) was dissolved in DMSO and administered at a concentration of 10 mg/kg via intraperitoneal injection once daily, as well starting 2 days before until 14 days post-crush. Controls received an equal volume of solvent.

### Clinical assessment of nerve functionality by walking track analysis

Nerve functionality was assessed 2 days before as well as 7 and 14 days post-crush by walking track analysis and calculation of the sciatic functional index (SFI) as described elsewhere [29].

### Electrophysiology

Electrophysiology was essentially performed as described previously [30]. Nerve conduction velocities and compound muscle action potentials were determined at 14 days post-crush. Mice were anesthetized with a mixture of ketamine (100 mg/kg) and xylazine (10 mg/kg) and immediately placed on a heating plate (37 °C) to maintain constant body temperature. Stimulation of the sciatic nerve was performed by repetitively generated single pulses using monopolar 30 G needle electrodes until supramaximal stimulation was achieved. Compound muscle action potential was recorded at the plantar foot muscle with a needle electrode using a portable electrodiagnostic system (KeyPoint 4, Medtronic, Meerbusch, Germany). Nerve conduction velocity was calculated from the distance and the motor latency differences between proximal and distal stimulations.

### Tissue preparation

Following electrophysiology, mice were sacrificed via cervical dislocation. Sciatic nerves were carefully removed by only handling the most proximal end with forceps and cutting the nerve at its most distal end using scissors. For immunohistochemical applications, the nerves were immediately dipped in an isopentane bath immersed in liquid nitrogen for approximately 5 s. Then frozen nerves were placed in suitable cryomolds, covered with a cryo-embedding compound and placed on dry ice. Embedded nerves were stored at −80 °C. Longitudinal sections of 7-μm thickness were prepared in a cryostat chamber, and slides were air-dried for at least 1 hour before further processing or stored at −20 °C.

### Antibodies

The following antibodies were used and diluted in Antibody Diluent (Dako, Hamburg, Germany) as indicated: Rabbit anti-cAMP polyclonal antibody—1:50 (Merck-Millipore, Darmstadt, Germany); biotinylated goat anti-rabbit IgG—1:200 (Vector Laboratories, Peterborough, UK); DyLight 594 Streptavidin—1:200 (Vector Laboratories); rabbit anti-neurofilament L (NF-L)—1:1000 (Merck-Millipore); and Alexa Fluor 488 Goat Anti-Rabbit IgG—1:200 (Life Technologies).

### Immunofluorescence

Sections were post-fixed in 4 % paraformaldehyde for 20 min. After fixation, slides were washed 5 min in PBS and twice for 5 min in PBT (PBS + 0.1 % Triton X-100). Slides were incubated with blocking solution (10 % normal goat serum (*v*/*v*) in PBT) for 30 min at room temperature. For the detection of cAMP, primary antibody was applied and slides were incubated at 4 °C for 16 h. Slides were washed twice for 5 min in PBT, and biotinylated secondary antibody was applied and incubated at room temperature for 1 hour. Slides were washed twice for 5 min in PBT, and DyLight594-conjugated streptavidin was applied. If desired, co-staining with NF-L was performed. Slides were washed 2 × 5 min in PBT and incubated with anti-NF-L antibody at room temperature for 1 hour. Slides were washed twice for 5 min and incubated with Alexa Fluor 488-conjugated secondary antibody. Slides were washed 5 min in PBT and 5 min in PBS and mounted with Vectashield Hardset mounting medium containing DAPI (Vector Laboratories).

### Preparation of semi-thin sections

Nerves were fixed in 0.1 M cacodylate buffer containing 2.5 % glutaraldehyde and kept at 4 °C overnight. The fixative was discarded and replaced by washing buffer (0.1 M cacodylate + 3 % sucrose). Nerves were washed for 4 days at 4 °C. Washing was followed by incubation in an osmium tetroxide reagent for 3 h. Osmium tetroxide reagent was composed of one part 5 % potassium dichromate solution (pH 7.4), one part 3.4 % NaCl solution, and two parts 2 % osmium tetroxide solution (Sigma-Aldrich). Afterwards, samples were briefly washed in 0.1 M cacodylate buffer.

Samples were dehydrated in an ascending ethanol series (70 %; 96 %; ≥99.8 % undenatured ethanol) for 1 hour each. Following dehydration, samples were incubated in 250 μl propylene oxide (Sigma-Aldrich) in tightly closed containers for 1 hour at room temperature, then 1 hour in a 1:1 mixture of propylene oxide/epon (epoxy embedding medium kit; Sigma-Aldrich), and finally kept at 4 °C in epon only overnight. Samples were placed in silicone molds and covered with epon embedding mixture. Embedded samples were incubated at 37 °C for 6 h, at 47 °C for 15 h, and finally at 60 °C for 28 h until epon was completely hardened. Transverse sections were prepared at a thickness of 1 μm at a Reichert-Jung Ultracut Microtome and immediately stained with toluidine blue (1 % toluidine blue (*w*/*v*) dissolved in a 1 % disodium tetraborate (*w*/*v*) solution), washed in distilled H_2_O (approximately 10 ml) containing 1–2 drops of acid ethanol (0.01 % HCl in absolute ethanol), placed on a microscope slide, dried on a heating plate, and mounted with Roti Histokitt II (Roth).

### Quantification of LPA by liquid chromatography coupled to tandem mass spectrometry (LC-MS/MS)

Sciatic nerve homogenates were extracted using 800 μl of a mixture methanol:chloroform (2:1) (both VWR International, Darmstadt, Germany) after addition of 20 μl LPA 17:1 (1 μg/ml) (Avanti Polar Lipids, Alabaster, AL, USA) as internal standard (IS), 50 μl deionized water (VWR), and 40 μl 6 M HCl (Merck, Darmstadt, Germany). Samples were vortexed for 5 min and incubated 20 min at -20 °C. After centrifugation (10 min, 14,000×*g*), the upper phases were transferred to new sample tubes and extracted with 200 μl chloroform (Merck) and 250 μl deionized water. Samples were vortexed rigorously and centrifuged for 10 min at 14,000×*g*. The lower organic phases were dried under nitrogen gas flow at 60 °C. The dried residues were dissolved in 50 μl LC-MS grade methanol (mobile phase B). Analysis was performed on a Waters/Acquity ultra performance LC-MS system controlled with MassLynx software. Analysis was operated with electrospray ionization (ESI) probe in negative ESI employing multiple reaction monitoring (mrm) mode. LPA species for the establishment of mrm experiments were obtained from Avanti Polar Lipids. The following transitions were used: m/z 435 [M-H-] to 153 (LPA 18:1), m/z 409 [M-H-] to 153 (LPA 16:0), m/z 437 [M-H-] to 153 (LPA 18:0), and m/z 421 [M-H-] to 153 for the internal standard (LPA 17:1). Collision energy (ce) and cone voltage (cv) were both set to 30 V for all mrm experiments. The column used for chromatography was a Thermo Scientific Hypersil Gold, 1.9 μm, 50 × 2.1 mm column. A total flow rate of 0.4 ml/min was applied with an injection volume of 10 μl. The mobile phases consisted of 5 mM ammonium formate (Sigma-Aldrich) buffered water (A) and methanol (B), both containing 0.1 % formic acid (Sigma-Aldrich). Gradient elution was performed starting at 50 % mobile phase B applying isocratic conditions for 2.5 min and then progressing to 100 % B over 3 min. 100 % B was held up for 2 min. Within 1 min, the gradient increased linear to initial conditions and was held up to 10 min for re-equilibration. Column temperature was adjusted at room temperature (25 °C ± 5 °C). The autosampler operated at 10 °C. Run time was set to 10 min. Retention times of LPA species were 2.99 min (LPA 18:1), 2.95 min (LPA 18:0), 3.10 min (LPA 16:0), and 2.17 min (LPA 17:1 IS), respectively. Data analysis was performed using peak-area-ratios of analytes to internal standard.

### Determination of nerve cytokine levels by ELISA

For the detection of cytokines, commercially available ELISA kits (Duo Set mouse TNF-α, R&D Systems, Minneapolis, MN, USA; Mouse IL-10 ELISA MAX Standard Set, Biolegend, San Diego, CA, USA) were used essentially as instructed. Briefly, sciatic nerves were homogenized in 250 μl ice-cold PBS containing cOmplete™ Mini (Roche, Basel, Switzerland) proteinase inhibitor cocktail. Homogenates were centrifuged for 10 min at 11,000 rpm, and supernatants were transferred to new sample tubes, diluted 1:2 with assay buffer (PBS containing 1 % BSA and 0.1 % Tween20) and vortexed rigorously. Detection of cytokines was performed as recommended by the manufacturers. Sciatic nerve cytokine content was normalized to the respective protein content of the sample.

### Image and data analysis

Analysis of images was performed using ImageJ (National Institutes of Health, Bethesda, MA, USA). Data analysis and compilation of graphs was performed using Microsoft (Redmond, WA, USA) Excel and GraphPad (La Jolla, CA, USA) Prism 5. Statistical analysis was done by Student’s *t* test, multiple comparisons were performed by one-way ANOVA followed by the Newman-Keuls post hoc test or Kruskal-Wallis test and Mann-Whitney *U* test for non-Gaussian distributions. Statistical significance is indicated by asterisks with *P* ≤ 0.05*, *P* ≤ 0.01**, and *P* ≤ 0.001***.

## Results

### Impact of fingolimod on electrophysiological and clinical measures after injury

We performed sciatic nerve crush in immunocompetent wildtype and in immunodeficient T- and B-lymphocyte deficient *Rag1*^*−/−*^ as well as in T-lymphocyte deficient *Foxn1*^*−/−*^ mice to identify lymphocyte-independent effects of fingolimod on peripheral nervous system regeneration.

**Fig. 1:**
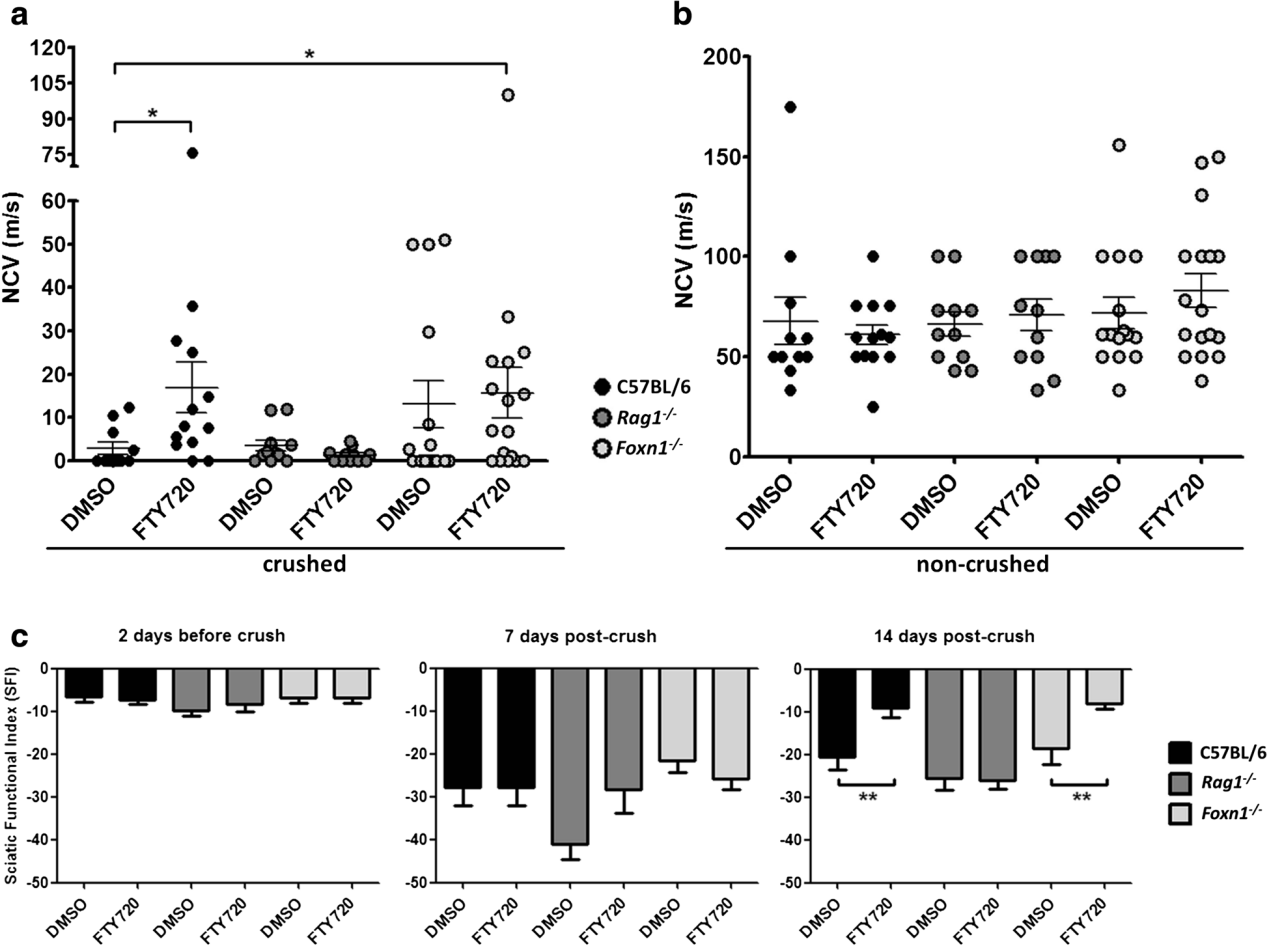
Fingolimod (FTY720) improves peripheral nerve regeneration by electrophysiological and clinical measures. **a** C57BL/6 as well as *Foxn1* ^*−/−*^ mice treated with fingolimod were found to display significantly improved nerve conduction velocities (NCV) compared to wildtype controls. *Rag1* ^*−/−*^ mice did not show an improvement of nerve regeneration by this measure. Statistical analysis was done by Kruskal-Wallis test and Man-Whitney *U* test. *N* = 11/13/11/11/15/17 animals (from left to right). **b** Nerve conduction was normal in contralateral non-crushed nerves of the same animals. **c** Assessment of functional recovery was determined by walking track analysis via sciatic functional indices (SFI). Two days before crush sciatic nerve function was normal in all three mouse strains. Seven days post-crush nerve function was significantly impaired in all three mouse strains (*P* ≤ 0.01) as indicated by a decline in SFI. Note that *Rag1* ^*−/−*^ control mice showed an additional mean, but non-significant worsening of nerve function. At 14 days post-crush, significantly improved nerve conduction is consistent with significantly improved SFI in fingolimod-treated C57BL/6 and *Foxn1* ^*−/−*^ animals. No significant difference in SFI was observed in *Rag1* ^*−/−*^ mice at this stage. *N* = 8/9/7/6/9/14 animals minimum for each graph (from left to right). Statistical analysis was done by Student’s *t* test, two-tailed. Data represent mean ± s.e.m. Statistical significance is indicated by asterisks with *P* ≤ 0.05*, *P* ≤ 0.01**, and *P* ≤ 0.001***

Fingolimod treatment resulted in significantly increased nerve conduction at 14 days post-crush in wildtype C57BL/6 mice (Fig. 1a). This finding is consistent with significantly improved functional recovery as determined by walking track analysis and SFI (Fig. 1c). In contrast, *Rag1*^*−/−*^ mice did not show an improvement of nerve regeneration by clinical or electrophysiological measures neither with fingolimod nor DMSO-only treatment (Fig. 1a, c), and DMSO-treated control Rag1^−/−^ mice even exhibited some additional mean, however, non-significant decline in SFI (Fig. 1c). However, *Foxn1*^−/−^ mice, which are devoid of T- but not B-lymphocytes, did show an improvement of nerve regeneration under fingolimod treatment (Fig. 1a, c). Although the mean increase in nerve conduction velocity in both fingolimod-treated and control *Foxn1*^*−/−*^ mice implies a potentially positive role of T-lymphocyte deficiency on nerve regeneration, only fingolimod-treated *Foxn1*^*−/−*^ mice showed a significant improvement compared to C57BL/6 controls and performed better in the functional analysis (Fig. 1a, c).

### Effect of fingolimod on axonal cAMP levels

**Fig. 2:**
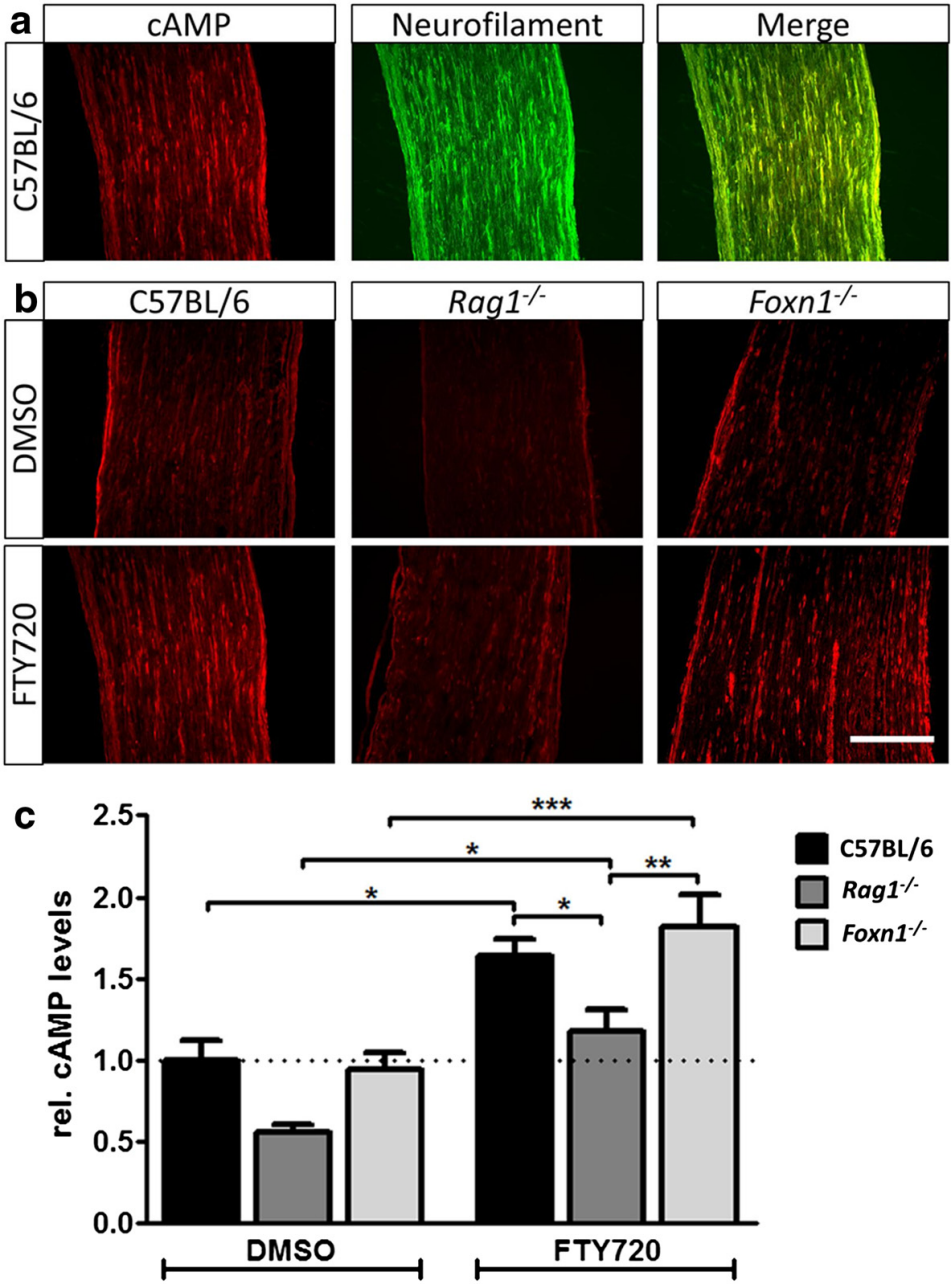
Fingolimod (FTY720) significantly elevates cAMP levels in the regenerating nerve. **a** Fourteen-day post-crush, colocalization of cAMP and neurofilament light-chain immunohistochemistry indicates an accumulation of axonal cAMP around and proximal from the injury site. **b** Representative images of cAMP immunofluorescence. *Scale bar* indicates 100 μm. **c** Quantification of cAMP immunofluorescence revealed significantly increased axonal cAMP levels in crushed nerves under fingolimod treatment in wildtype mice and both immunodeficient mouse strains. Note that although fingolimod induced a significant cAMP increase in *Rag1* ^*−/−*^ mice compared to *Rag1* ^*−/−*^ controls, overall cAMP levels were reduced in *Rag1* ^*−/−*^ mice, which correlates with the impairment of regenerative capacity in these mice. *N* = 4/4/6/4/4/6 animals (from left to right; quantification of one representative slide per animal). Statistical analysis was done by one-way ANOVA and Newman-Keuls post hoc test. Data represent mean ± s.e.m. Statistical significance is indicated by asterisks with *P* ≤ 0.05*, *P* ≤ 0.01**, and *P* ≤ 0.001***

To better understand potentially relevant molecular mechanisms by which fingolimod may exert the observed neuroregenerative effects, we investigated cAMP, an axonal outgrowth enhancing factor that accumulates proximal from and around the injury site during axonal regeneration [31–34]. We hypothesized that fingolimod causes abrogation of S1P_1_-mediated signaling through S1P_1_ receptor internalization in cells of the peripheral nerve, thereby indirectly increasing cAMP levels by reduced inhibition of adenylate cyclase. Quantification of cAMP immunofluorescence proximal from and around the injury site at 14 days post-crush revealed significantly increased cAMP levels under fingolimod treatment in C57BL/6 and *Foxn1*^*−/−*^ mice (Fig. 2a–c), and this increase in cAMP paralleled significantly improved clinical and electrophysiological measures in these animals (Fig. 1). In contrast, *Rag1*^*−/−*^ mice did not show a relevant elevation of cAMP levels under fingolimod treatment when compared to wildtype or *Foxn1*^*−/−*^ controls and cAMP levels were even significantly reduced in fingolimod-treated *Rag1*^*−/−*^ compared to fingolimod-treated C57BL/6 and *Foxn1*^*−/−*^ mice. Strikingly, overall reduced axonal cAMP correlates with impaired regeneration in fingolimod-treated *Rag1*^*−/−*^ mice.

### Myelin thickness after injury and inhibition of autotaxin

**Fig. 3:**
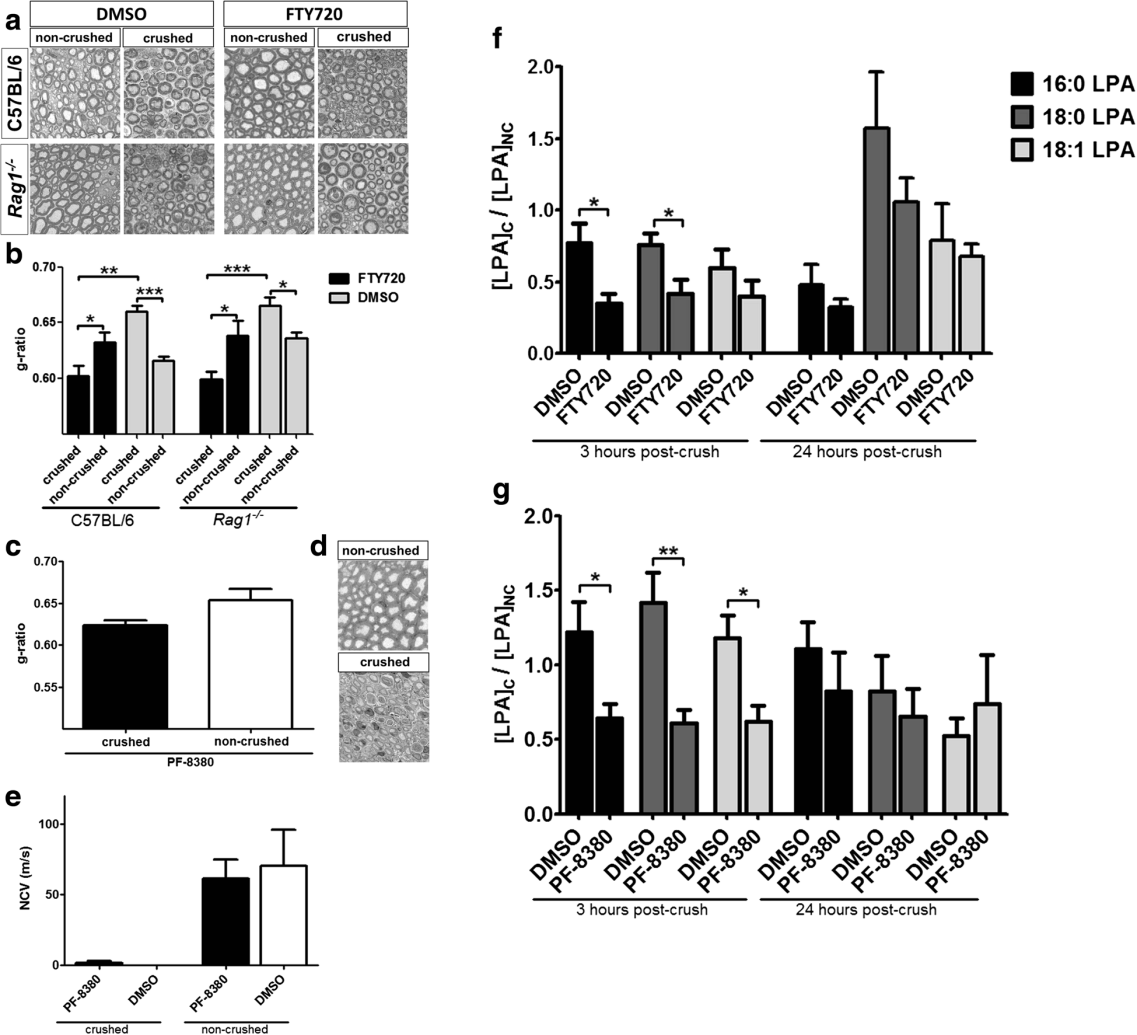
Fingolimod (FTY720) enhances myelin thickness independent of its immunomodulatory properties. **a** Representative images of semi-thin sections used for g-ratio measurements. **b** G-ratios indicate significantly reduced myelin thickness in crushed nerves of C57BL/6 and *Rag1* ^*−/−*^ control animals compared to myelin thickness of contralateral non-crushed nerves. Fingolimod-treated mice showed a reciprocal ratio, with slightly, but even significantly increased myelin thickness in crushed nerves compared to contralateral non-crushed nerves. *N* = 5/5/4/4/4/4/4/4 animals (from left to right, quantification of one slide per animal). **c** Treatment of C57BL/6 mice with the autotaxin inhibitor PF-8380 prevented the significant reduction in myelin thickness in regenerating axons normally observed after crush injury (*N* = 4/4 animals, quantification of one slide per animal). **d** Representative images of semi-thin sections used for g-ratio measurements at 14-day post-crush in PF-8380 treated mice. **e** PF-8380 treated C57BL/6 mice did not show an improvement in nerve conduction at 14-day post-crush, pointing to no effect of autotaxin inhibition on axonal regeneration (*N* = 4/4 animals). **f** Fingolimod significantly reduces sciatic nerve concentration of 16:0 and 18:0 LPA at 3-h post-injury in crushed nerves of C57BL/6 mice. LPA concentration is still reduced in average at 24-h post-crush. However, in DMSO-treated control mice, nerve injury did not induce invariably increased LPA levels in the crushed nerve compared to contralateral non-crushed nerves. LPA levels are depicted as a ratio calculated from LPA in the crushed nerve and the corresponding contralateral non-crushed nerve (LPA_c_/LPA_NC_) to account for individual LPA levels between different animals. (*N* = 4 for each column). **g** PF-8380 significantly reduces LPA levels at 3-h post-injury in crushed nerves of C57BL/6 mice (*N* = 5 animals for each column (3 h) or four animals for each column (24 h), respectively) (*C* crushed, *NC* non-crushed). Statistical analysis was done by Student’s *t* test, two-tailed. Data represent mean ± s.e.m. Statistical significance is indicated by asterisks with *P* ≤ 0.05*, *P* ≤ 0.01**, and *P* ≤ 0.001***

Following peripheral nerve injury, myelin sheaths of regenerated axons are commonly found to be significantly thinner compared to those of contralateral control nerves [35]. Therefore, to assess the effect of fingolimod on myelin thickness after injury, we performed g-ratio (the numerical ratio between the diameter of the axon and the diameter of the outer myelinated fiber) measurements from semi-thin sections prepared from the distal stump of injured and control nerves at 14 days post-crush (Fig. 3a). Analysis of g-ratios revealed significantly decreased myelin thickness in crushed nerves of control animals compared to myelin thickness in contralateral non-crushed nerves whereas myelin thickness was significantly increased in crushed nerves of fingolimod-treated mice (Fig. 3b). This was also the case in *Rag1*^*−/−*^ mice. Therefore, we hypothesized that fingolimod may improve myelin thickness by diminishing LPA levels in the injured nerve via inhibition of autotaxin. We assessed LPA levels in the sciatic nerve of C57BL/6 mice at 3 and 24 h post-crush under DMSO and fingolimod treatment. Analysis of sciatic nerve LPA content by LC-MS/MS revealed significantly reduced LPA levels at 3 h post-crush in fingolimod-treated mice (Fig. 3f). Twenty four hours post-crush LPA levels were still lower in average, though not significantly. However, LPA levels were not found to be invariably increased in the crushed nerves of DMSO-treated control mice, indicating that physiological LPA levels produced by autotaxin may be sufficient to induce demyelination after injury. To evaluate the functional consequence of LPA reduction, we assessed myelin thickness in C57BL/6 mice treated with the specific autotaxin inhibitor PF-8380. G-ratio measurements from semi-thin sections obtained at 14 days post-crush revealed normal and strikingly not significantly reduced myelin thickness in distal regenerating axons (Fig. 3c), pointing to a role of LPA in demyelination or downregulation of myelin proteins, respectively. Finally, to distinguish LPA from S1P-mediated effects on axonal regeneration, we performed electrophysiology with animals treated with PF-8380 at 14 days post-crush. We could not detect any signs of improved nerve conduction in these mice, suggesting that inhibition of autotaxin and lowering LPA levels, respectively, may primarily affect myelination, but not peripheral axon regeneration (Fig. 3e).

**Fig. 4:**
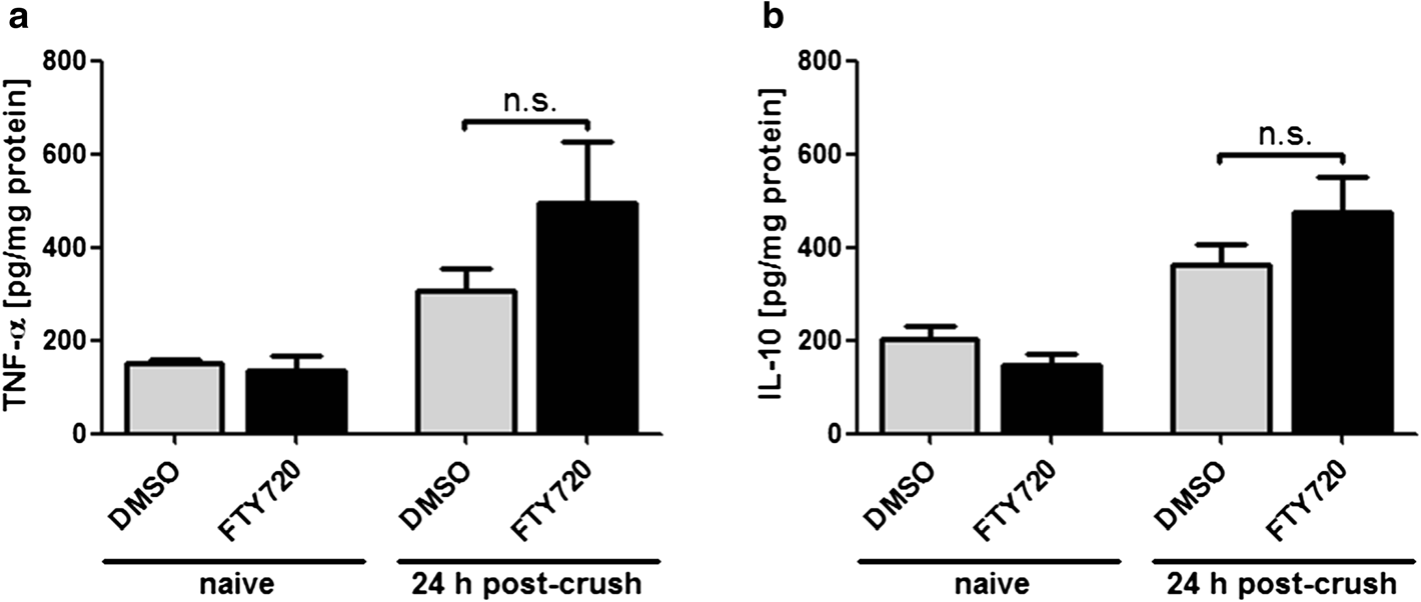
Fingolimod (FTY720) does not alter pro- and anti-inflammatory cytokine levels after injury. Neither sciatic nerve levels of TNF-α (**a**) nor IL-10 (**b**) were found to be significantly affected by fingolimod treatment at 24 h post-crush. Baseline sciatic nerve cytokine levels were determined in naive mice. *N* = 3/3/11/9 animals for each graph, from left to right. Statistical analysis was done by Student’s *t* test, two-tailed; n.s. indicates no significant difference. Data represent mean ± s.e.m

### Sciatic nerve cytokine levels after injury

Since cytokines are known to potentially impact nerve regeneration [36, 37], we finally tested whether fingolimod affects sciatic nerve levels of the pro-inflammatory cytokine tumor necrosis factor alpha (TNF-α) and the anti-inflammatory cytokine interleukin 10 (IL-10), which are both known to be highly expressed at 24-h post-injury [38]. We recognized a significant increase of both TNF-α and IL-10 levels after injury but could not find fingolimod to significantly affect this cytokine response (Fig. 4).

## Discussion

In this work, we provide in vivo evidence for direct effects of the S1P receptor agonist fingolimod on the peripheral nervous system that may result in the promotion of peripheral nerve regeneration. Although certain positive effects of fingolimod-induced T-lymphocyte sequestration on nerve regeneration cannot be ruled out completely, our data strongly indicate that direct effects of fingolimod on neurons and Schwann cells are responsible for improved nerve regeneration. Fingolimod treatment was associated with a significant elevation of axonal cAMP, a crucial factor for central and peripheral axon regeneration [31–34]. The observed increase in cAMP may most likely be the result of fingolimod-induced S1P_1_ receptor internalization in cells of the peripheral nerve that may cause the abrogation of S1P_1_-mediated inhibition of adenylate cyclase. It has been proposed that the S1P_1_-G_i_-adenylate cyclase system may become internalized as a ternary complex, leading to sustained inhibition of adenylate cyclase as long as the ligand fingolimod-P is bound [39]. However, we propose differences between “short-term” (several hours) and “long-term” (several days to weeks) administration of fingolimod as, in contrast to inhibition of cAMP formation in cell culture experiments by sustained signaling from intracellular compartments [40], our data demonstrate that long-term administration of fingolimod in vivo may result in an elevation of cAMP. The internalized S1P_1_-G_i_-adenylate cyclase system is likely to become degraded over time, and constantly high concentrations of fingolimod-P may prevent the localization of de novo synthesized S1P_1_ to the plasma membrane. Hence, the pivotal difference in the long-term situation may be the spatial segregation of S1P_1_ and adenylate cyclase, consequently allowing for an increased cAMP response in the regenerating nerve. Clearly, further studies are needed to corroborate this assumption.

In contrast to T-lymphocyte-deficient *Foxn1*^*−/−*^ mice which exhibited signs of significantly improved nerve regeneration under fingolimod treatment, B- and T-lymphocyte deficient *Rag1*^*−/−*^ mice did not show an improvement of nerve regeneration, neither with fingolimod nor vehicle treatment. Although one recent study has reported evidence for improved nerve regeneration after femoral nerve injury in *Rag2*^*−/−*^ mice which lack functional T- and B-lymphocytes as well [41], others have found nerve regeneration and motor neuron survival to be reduced in *Rag2*^*−/−*^ mice [42], which is in line with our observations in *Rag1*^*−/−*^ mice. The idea of an overall reduced regenerative capacity of *Rag1*^*−/−*^ mice is further supported by the finding that B-lymphocytes appear to be required for the production of autoantibodies against myelin debris containing inhibitors of axonal outgrowth, consequently allowing for normal axonal regeneration after nerve injury [43]. Myelin-associated inhibitors of axonal outgrowth have been implicated in reducing neuronal cAMP in a G_i_-dependent manner via activation of the Nogo receptor-p75 neurotrophin receptor complex [44], providing a likely explanation for significantly reduced axonal cAMP in response to fingolimod treatment and, consistently, the impaired regenerative capacity observed in *Rag1*^*−/−*^ mice due to the lack of B-lymphocytes. Although fingolimod is known to also reduce the number of circulating B-lymphocytes, it has recently been shown to increase the proportion of regulatory B-lymphocytes producing IL-10. While we could not detect a significant elevation of IL-10 nor a decrease in TNF-α levels in the sciatic nerve of fingolimod-treated mice at 24-h post-crush (Fig. 4), an increase in IL-10 secreting B-lymphocytes may occur at later stages and could potentially contribute to accelerated nerve regeneration [37, 45].

However, despite decreased cAMP, *Rag1*^*−/−*^ mice still showed significantly improved myelin thickness. Another member of the lysophospholipid family, LPA, was found to be significantly reduced in the crushed nerve in fingolimod-treated mice shortly after injury. Strikingly, inhibition of autotaxin by the specific inhibitor PF-8380 prevented the significant reduction of myelin thickness normally observed after peripheral nerve injury. Although LPA levels were not invariably and not significantly increased in control mice, it appears unlikely that a sole increase in LPA would account for demyelination, but rather an elevated expression of the LPA_1_ receptor after injury. In this context, it has been reported that the LPA_1_ receptor is upregulated within the first days after sciatic nerve injury in the distal nerve stump, and this upregulation is accompanied by an abrupt downregulation of the myelin gene *P0* [46]. In line with this, a single intrathecal injection of LPA or nerve injury was found to cause a decrease in the expression of the myelin proteins PMP22 and MBP, but the demyelinating effect was largely abolished in LPA_1_-null mice [19]. Furthermore, antagonism of the LPA_1_ receptor has recently been demonstrated to reduce demyelination after spinal cord injury [21]. Therefore, improved myelination could be a consequence of both fingolimod as well as PF-8380-mediated inhibition of autotaxin, attenuating LPA_1_ receptor activation via reduced LPA synthesis. Although autotaxin represents only one of multiple metabolic pathways to produce LPA, autotaxin appears to be one major contributor to LPA synthesis after injury, as it was previously shown that injury-induced dorsal root demyelination is significantly reduced in heterozygous autotaxin knockout mice [19]. However, since PF-8380 treatment did not lead to signs of improved axonal regeneration, our results might suggest a dual mode of action for fingolimod in peripheral nerve regeneration—fingolimod may promote axonal regeneration by indirectly elevating cAMP formation as a consequence of S1P_1_ receptor internalization and may prevent LPA-induced downregulation of myelin gene expression in the regenerating nerve. Further studies are warranted to corroborate this assumption.

## Conclusions

Collectively, our data presented here demonstrate that modulation of lysophospholipid signaling in the peripheral nervous system by fingolimod may enhance nerve regeneration by acting on multiple molecular and cellular levels and partly independent of its anti-inflammatory effects. In addition to the established role of fingolimod as a modulator of S1P receptor-mediated signaling, the inhibition of LPA synthesis after injury may represent a yet unrecognized mechanism that contributes to the presumptive remyelinating effect of fingolimod.

## Abbreviations

cAMP, cyclic adenosine monophosphate; *Foxn1*, forkhead box protein N1; IL-10, interleukin 10; LPA, lysophosphatidic acid; LPA_1_, lysophosphatidic acid receptor 1; NF-L, neurofilament light chain; *Rag1*, recombination activating gene 1; S1P, sphingosine-1-phosphate; S1P_1_, sphingosine-1-phosphate receptor 1; SFI, sciatic functional index; TNF-α, tumor necrosis factor alpha.
